# Diabetic Nephropathy: a Tangled Web to Unweave

**DOI:** 10.1007/s10557-017-6755-9

**Published:** 2017-09-27

**Authors:** Corey Magee, David J. Grieve, Chris J. Watson, Derek P. Brazil

**Affiliations:** Centre for Experimental Medicine, Queen’s University Belfast, Wellcome-Wolfson Building, 97 Lisburn Road, Belfast, Northern Ireland BT9 7AE UK

**Keywords:** Diabetic nephropathy, Vascular dysfunction, Renin-angiotensin-aldosterone system, Oxidative stress, Inflammation, Novel therapies

## Abstract

Diabetic nephropathy (DN) is currently the leading cause of end-stage renal disease globally. Given the increasing incidence of diabetes, many experts hold the view that DN will eventually progress toward pandemic proportions. Whilst hyperglycaemia-induced vascular dysfunction is the primary initiating mechanism in DN, its progression is also driven by a heterogeneous set of pathological mechanisms, including oxidative stress, inflammation and fibrosis. Current treatment strategies for DN are targeted against the fundamental dysregulation of glycaemia and hypertension. Unfortunately, these standards of care can delay but do not prevent disease progression or the significant emotional, physical and financial costs associated with this disease. As such, there is a pressing need to develop novel therapeutics that are both effective and safe. Set against the genomic era, numerous potential target pathways in DN have been identified. However, the clinical translation of basic DN research has been met with a number of challenges. Moreover, the notion of DN as a purely vascular disease is outdated and it has become clear that DN is a multi-dimensional, multi-cellular condition. The review will highlight the current therapeutic approaches for DN and provide an insight into how the inherent complexity of DN is shaping the research pathways toward the development and clinical translation of novel therapeutic strategies.

## Introduction

Diabetes mellitus (DM) is a systemic disease that is characterised by an inability of the body to either produce or effectively respond to the glucose-regulating hormone, insulin [1]. Type 1 DM results from an idiopathic autoimmune destruction of pancreatic β-cells, whilst widespread peripheral insulin resistance drives type 2 DM [1]. The resulting hyperglycaemia upsets haemodynamic and metabolic homeostases, whilst the chronic nature of this microenvironmental disequilibrium promotes the development of diffuse cellular abnormalities. As a result, many patients develop serious complications, the most devastating of which is extensive vascular dysfunction [2]. Given that the kidneys are highly sensitive to both haemodynamic and metabolic alterations, these organs are vulnerable targets within the diabetic milieu. Diabetic nephropathy (DN) is a key microvascular complication of DM. DN is characterised by albuminuria (urinary albumin to creatinine ratio ≥ 30 mg/g) and an eventual decline in the estimated glomerular filtration rate (eGFR < 60 ml/min/1.73 m^2^) [3]. DN follows distinct phases, where glomerular hyperfiltration is followed by a relentless decline in renal function, typically occurring over a 15–20 year period.

Currently, DN is the leading cause of end-stage renal disease (ESRD) globally [3]. Whilst the natural history of DN can vary slightly, patients often experience a chronic condition where progressive structural changes in the kidney correlate with various stages of clinical renal deterioration. The development of ESRD requires the use of dialysis or renal transplantation, both of which can be associated with excess morbidity and mortality [4, 5]. Moreover, DN incidence rates show no signs of slowing. In the USA alone, 42% of all ESRD cases had a diagnosis of DN [6]. The importance of maintaining optimal renal function is further evidenced by the fact that DN is responsible for the vast proportion of excess mortality risk in patients with DM [7, 8]. Thus, there is a clear unmet need to develop novel DN-targeted therapeutics that can curtail the unrelenting progression of this disease and, hence, improve both the quality and quantity of life for this patient group. This review will highlight the key molecular mechanisms that drive DN whilst stressing the need to view DN as more than a simple vascular complication. More importantly, it will provide a critical insight into a range of exciting new DN treatment strategies that are on the horizon.

## Diabetic Nephropathy Pathogenesis

### Metabolic and Haemodynamic Dysregulations

**Fig. 1:**
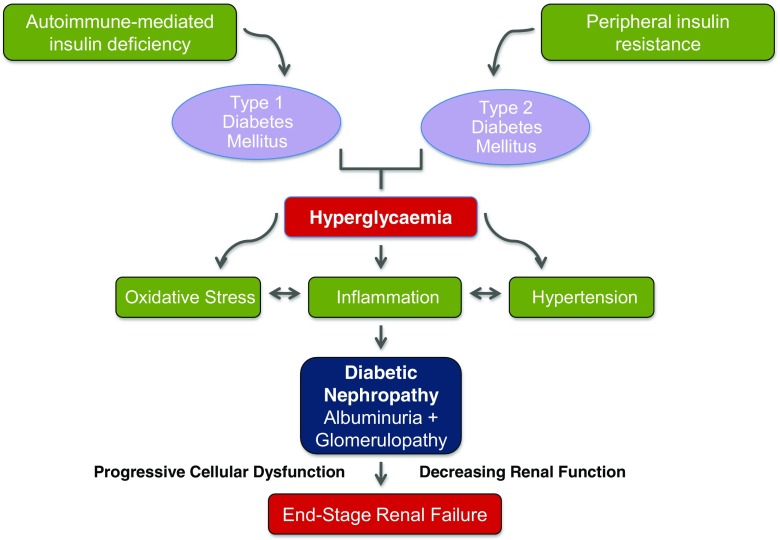
The central role of hyperglycaemia in the development and progression of diabetic nephropathy

DN pathogenesis is characterised by a complex interplay between metabolic and haemodynamic disruptions [9]. In DN, hyperglycaemia-induced metabolic impairment is central to the development and progression of DN (Fig. 1) and, hence, the appearance of defining renal lesions [10]. These include glomerular basement membrane thickening, mesangial expansion and the appearance of characteristic Kimmelstein-Wilson nodules. These changes occur secondary to hyperglycaemia-induced oxidative stress and accumulation of advanced glycation end products. Additional critical mediators of DN include haemodynamic elements, such as activation of vasoactive hormonal pathways, most commonly the renin-angiotensin-aldosterone system (RAAS). The associated systemic hypertension contributes to increased intra-glomerular pressure [8]. Glomerular microvascular lesions develop secondary to altered blood flow and increased vascular permeability. The underlying signalling pathways involved are highly complex, and current research is intensively focused on a range of key molecular drivers.

Central to aberrant signalling in DN is the transforming growth factor-beta 1 (TGF-β_1_), which has long been recognised as a key driver of DN [11]. TGF-β_1_ expression is elevated within various constituent kidney cells in DN [12, 13]. Dysfunctional TGF-β_1_ signalling modulates glucose flux into renal cells by upregulating the glucose transporter, GLUT-1 [10]. In effect, TGF-β_1_ is at the centre of a complex signalling cascade-driving DN [14]. SMAD proteins are the intra-cellular signalling effectors of TGF-β_1_ secondary to their ability to translocate to the nucleus and regulate transcription [15]. Animal models have highlighted the importance of the TGF-β/SMAD pathway in the development of diabetic glomerulosclerosis and tubulointerstitial fibrosis [16, 17]. Beyond this canonical pathway, recent studies have proven the existence of complex crosstalk between TGF-β and other well-known SMAD-independent pathways, such as Src proto-oncogene non-receptor tyrosine kinase (Src) signalling. Moreover, this redox-driven crosstalk has been shown to upregulate pro-fibrotic markers [18] and may provide new opportunities for targeted intervention.

Aside from the TGF-β pathway, a host of newly implicated genes has been identified, due in part to the upsurge in genome-wide association studies (GWAS). These have proven to be powerful tools for defining the genetic architecture of DN in patients. However, limitations of the genome-wide approach were recently exemplified by the GENIE consortium [19]. Despite being the largest DN-focused GWAS to date, this study highlighted the vast complexity underlying DN heritability. It is also important to realise that many newly identified genes likely only account for a limited proportion of the observed phenotypic alterations in patients. Furthermore, it is possible that the pathogenic pathways differ between type 1- and type 2-associated DN. Additionally, there is a distinct likelihood that unique mechanisms promote disease initiation versus disease progression.

Given that metabolic and haemodynamic changes are central pathogenic drivers of DN, approaches to target these mechanisms have become standards of care. However, it has become clear that the management of hypertension [20] and the pursuit of tight glycaemic control [21] are ineffective in stopping the inevitable renal deterioration in many patients. As a result, researchers are continuing to advance the understanding of DN, and as such, a wealth of novel therapeutic targets are being identified. Such developments will be essential to facilitate more effective diagnosis and treatment of DN.

### Diabetic Nephropathy: a Multi-cellular Affair

By virtue of the complex anatomical structure and intricate physiology of normal kidneys, the intrinsic pathological features of DN involve a multitude of constituent renal cells. As highlighted previously, aberrant cellular signalling promotes characteristic histological changes within diabetic kidneys via the release of numerous secreted factors [22]. When attempting to rank dysfunction of specific cellular types, DN has often been viewed as a ‘podocentric’ disease, where the highly specialised podocytes are often described as the ‘weakest link’ among the renal cell population [22]. However, glomerular endothelial cells (GEnCs) and tubulointerstitial cells are increasingly implicated in the development and progression of DN [23–26].

A common feature of diabetic cardiovascular complications is diffuse endothelial injury, and DN is no exception. GEnCs are continuous, highly fenestrated cells that are covered by a thick glycoprotein layer of glycocalyx. It has been demonstrated that altered permeability of the GEnC layer can exacerbate levels of albuminuria, independent of ultrastructural changes in the podocytes [27]. Interestingly, in a study by Qi et al. which compared the transcriptomes of DN-resistant mice versus DN-susceptible mice, there was a significant downregulation of mitochondrial gene expression in the GEnCs of diabetic mice, which was associated with an increase in mitochondrial DNA lesions [24]. These results were confirmed using human tissue samples, where the authors correlated their findings with rapidly progressing DN [24]. These data provide new insights into the complexity of GEnC pathology in DN and align with the unifying concept that oxidative stress is central to diabetic vascular disease.

The myofibroblast represents another principal mediator of DN. These mesenchymal cells promote extracellular matrix accumulation and, hence, correlate positively with the amount of renal fibrosis. Whilst their role in disease progression has been firmly established, their origin remains controversial [28]. One explanation that has gained attention centres on the process of epithelial-to-mesenchymal transition (EMT). During EMT, renal epithelial cells undergo phenotypic changes, involving loss of epithelial markers and increased expression of mesenchymal genes [29]. Indeed, immunofluorescence has confirmed the presence of cells that stained positive for α-smooth muscle actin (α-SMA), a smooth muscle differentiation marker expressed by myofibroblasts, in renal interstitial tissue originating from type 2 DM patients [30]. Such findings directly contrast with work published by Humphreys et al. who implemented Cre/Lox techniques to genetically label renal epithelial cells in a mouse model of renal fibrosis [31]. By using either β-galactosidase or red fluorescent protein as markers to track the fate of renal epithelial cells in vivo, Humpheys et al. found no evidence to support the interstitial migration of epithelial cells, highlighting a notable lack of α-SMA-positive tubular cells [31]. This group suggested that the myofibroblast arises from the vascular pericyte and, hence, that therapeutic strategies against fibrotic kidney disease should target pericyte differentiation [31].

Recent papers from the Nieto and Kalluri groups have demonstrated a key role for the Snail transcription factor in kidney fibrosis and suggest that epithelial cells undergo a ‘partial’ EMT without continuing to the myofibroblast population [32, 33]. By the same token, the role of an analogous process called endothelial-to-mesenchymal transition (EndoMT) in DN is beginning to become clear. Evidence by Li et al. demonstrated the existence of this phenomenon in streptozotocin (STZ)-induced diabetic mice, a type 1 diabetic model [34], whilst a 2017 paper by Shang et al. extended this process to human samples by showing that nucleotide-binding oligomerization domain-containing protein 2 (NOD2) promotes EndoMT in DN biopsy tissue contributing to renal fibrosis [35]. Furthermore, recent publications have highlighted a novel role for bone marrow-derived myofibroblasts in renal fibrosis showing that pro-inflammatory macrophages are a source of collagen-producing myofibroblasts in both experimental and human models of DN [36, 37].

Consideration of any cell type in isolation is limited, as interactions between these cells are essential under both physiological and pathological conditions. In this way, there is a continuous bidirectional crosstalk that involves the exchange of numerous cytokines and growth factors. Moreover, the phenotypic and functional heterogeneities among renal cells, in particular renal endothelial cells, could imply the involvement of numerous unique signalling pathways. As such, it is now clear that the pathophysiology underpinning DN is a complex multi-molecular, multi-cellular process and therefore, targeting therapeutic strategies around more than one of these pathological targets may be a sensible approach.

### Epigenetics: the Missing Piece of the Puzzle?

For years, researchers have focused on the pivotal role of hyperglycaemia in the development of diabetic vascular complications. However, it is known that DN can develop even in those patients who show optimal blood glucose control [38, 39]. Moreover, the decline in renal function among DN patients is highly variable. In this regard, epigenetics is emerging as a next-generational paradigm to characterise DN. Epigenetics refers to the regulation of gene expression and phenotype that occurs without the need for DNA sequence changes. In keeping with the multi-factorial aetiology of DM, it is clear that environmental factors and health behaviours influence disease pathology by inducing changes in epigenetically regulated mechanisms in chromatin, including DNA methylation and histone post-translational modifications [40].

DNA methylation involves a methyl group being added to the fifth carbon position of cytosine [41], and as a result, this process inversely regulates gene expression. DNA methylation is an important modification in the context of complex diseases. Altered DNA methylation profiles were detected in DN [42, 43], and such investigations have documented differential methylation of various mediators of glomerular cell apoptosis [41]. Furthermore, genome-wide methylation screening has identified genes that are selectively hypermethylated in fibrotic fibroblasts and thus, DNA methylation has been strongly implicated in fibroblast-induced renal fibrosis [44]. Bechtel et al. highlighted that DNA methylation changes regulate the actions of TGF-β_1_ and that hypermethylation of RAS protein activator like 1 (RASAL1) correlated strongly with renal fibrogenesis [44].

Another compelling field of research is the role of non-coding RNAs (ncRNAs) in DN pathogenesis. Despite their appeal as novel biomarkers, the function of ncRNAs, such as microRNAs, is somewhat perplexing. This is evidenced by investigations into the role of mir-192, which demonstrated both pro- and anti-fibrotic capabilities [45, 46]. Also, it is important to realise that microRNAs rarely act in isolation. This fact was nicely highlighted by a report that TGF-β_1_ indirectly regulated endoplasmic reticulum stress in diabetic mice, via targeting a mega-cluster of around 40 microRNAs [47]. The role of long non-coding RNAs (lncRNAs) in DN is now beginning to emerge. LncRNAs are ≥ 200 nucleotides in length and function by means other than coding for proteins. As such, plasmacytoma variant translocation 1 (PVT1) was associated with diabetic ESRD in both type 1 and type 2 DM [48]. Further work highlighted that PVT1 expression was upregulated by hyperglycaemia and that knockdown of this lncRNA reduced the levels of key extracellular matrix proteins in human mesangial cells [49]. RNA sequencing analysis of renal glomeruli identified differential expression of the lncRNA, taurine upregulated 1 (Tug1) in the diabetic milieu [50]. Recently, Li et al. showed that DN-associated upregulation of metastasis-associated lung adenocarcinoma transcript 1 (MALAT1) expression promoted renal tubular epithelial pyroptosis, an inflammatory form of programmed cell death, by amplifying the inflammatory response via modulation of ELAV-like RNA-binding protein 1 (EVAL1) signalling [51]. In the field of non-coding RNAs, it is expected that future work will uncover a plethora of targets associated with DN.

Histone deactylation is another component of epigenetic regulation, which converts permissive euchromatin to repressive heterochromatin and, hence, causes transcriptional silencing. Histone deacetylases (HDACs) have garnered recent attention due to their ability to effectively regulate the transcriptional network. Despite relatively few publications, it is likely that HDACs regulate key signalling events within the diabetic kidney. Evidence to support this hypothesis has been provided by both in vitro and in vivo research: For example, Noh et al. showed that HDAC-2 could promote extracellular matrix accumulation in both renal tubular epithelial cells and renal tissue of STZ-induced diabetic mice [52]. Increased expression of HDAC-2/4/5 was documented in renal tissues of diabetic animals and biopsy material from diabetic patients [53]. In particular, HDAC-4 was found to selectively exacerbate podocyte damage in DN [53].

Aside from histone modification, HDACs may regulate non-histone proteins. HDACs have thus been highlighted as potential regulators of pro-inflammatory gene expression in DM [54]. The ability of HDACs to interact with the innate and adaptive immune responses implicates these molecules in the inflammatory pathogenesis of the diabetic kidney. In particular, NLR family pyrin domain-containing 3 (NLRP3) and other inflammasome components act to trigger the inflammatory cascade in the diabetic kidney [55]. In keeping with this, activation of caspase-1 has been shown to downregulate sirtuin 1 [56], a class III HDAC that usually engages in anti-oxidative and anti-inflammatory functions. In fact, sirtuin 1 downregulation leads to endothelial dysfunction in DM [57]. Altered HDAC function may therefore contribute to a wide range of pathological processes in DN. In contrast, HDAC-6 has been shown to negatively regulate NLRP3 activation [58] and thus, therapeutic modulation of HDACs may emerge as a useful therapeutic approach in DN. Although the precise roles of HDACs remain poorly understood, their ability to regulate both histone and non-histone proteins suggests that future research will unearth pivotal roles in DN pathology.

To move a step further, it is now becoming clear that these epigenetic capabilities confer ‘memory’ onto the cells. This phenomenon of ‘metabolic memory’ or more specifically ‘hyperglycaemic memory’ is common to both macro- and microvascular diabetic complications. In essence, this means that even transient periods of suboptimal glucose control can result in long-lasting consequences. This was evidenced in numerous models by the persistence of oxidative stress [59] and a failure to resolve glomerular basement membrane thickening [60] following blood glucose normalisation. It appears that these cells may become imprinted with epigenetic marks that are difficult, if not impossible, to reverse.

Even though the details of this complex process remain elusive, research has uncovered some important contributors. In this regard, it was suggested that prolonged epigenetic changes, as well as the long-term accumulation of advanced glycation end products, are compatible with this concept [61]. Furthermore, Geraldes et al. recently identified a novel marker of podocyte-specific memory [62]. Having previously demonstrated increased expression of the protein tyrosine phosphatase, SHP-1, in the renal cortex of type 1 diabetic mice, this group subsequently showed that this molecule remained elevated in podocytes even after systemic glucose normalisation [62]. When considering the vast amount of published data, there can be no doubt that the diabetic microenvironment induces profound epigenetic changes and, hence, alters the fine balance between beneficial and pathogenic transcriptome regulators. Furthermore, the ability to modulate metabolic memory may represent a powerful tool for the treatment of DN and other diseases. By exploring the relatively uncharted epigenetic landscape, researchers may uncover important insights into the molecular basis of DN.

## Current Standards of Care

### Controlling Blood Glucose: the Holy Grail

For years, effective glycaemic control has been hailed as the principal approach for reducing the incidence of diabetic complications. In the setting of DN, the benefits of tight glucose control were highlighted by examining the effects of sustained normoglycaemia following pancreatic transplantation. Fioretto et al. observed a reversal of renal lesions in DN patients after 10 years of normoglycaemia [63]. These landmark results suggested that precisely controlled glucose levels had the potential to promote significant reverse remodelling within the diabetic kidney. A number of authoritative studies have continued to support such benefits, including the Diabetes Control and Complications Trial (DCCT) [64]. In its type 1 DM cohort, one DCCT demonstrated that intensive glucose control improved various surrogate end points, including macroalbuminuria [64]. Additionally, the follow-up stage Epidemiology of Diabetes Interventions and Complications (EDIC) trial identified long-lasting risk reductions among the intensive therapy arm even when tight glucose control was no longer in place [65]. Further encouraging results came from the United Kingdom Prospective Diabetes Study (UKPDS) where intensive glycaemic control achieved an 11% reduction in glycosylated haemoglobin (HbA1c), a marker of glycaemic control in established DM, as well as a 33% decrease in microalbuminuria levels when compared to conventional therapy at a median follow-up of 10 years [66]. Such results were replicated by the ADVANCE (Action in Diabetes and Vascular Disease: Preterax and Diamicron Modified Release Controlled Evaluation) trial, which observed a notable 65% risk reduction for ESRD in the intensively managed arm of the study [67]. These findings have far-reaching ramifications, as reductions in renal disease indices should presumably associate with a reduced overall cardiovascular risk profile. However, whilst albuminuria is a well-established hallmark of DN, its sensitivity is somewhat limited. In this way, daily intra-patient fluctuations can confound its analysis. The ACCORD (Action to Control Cardiovascular Risk in Diabetes) trial also identified discordance between albuminuria and eGFR [68]. The ACCORD trial further raised concerns regarding strict glycaemic management by highlighting excessive mortality in the intensive therapy arm [68]. In a similar manner, analyses from the ADVANCE trial identified an association between hypoglycaemia and the development of adverse clinical effects [67]. These findings highlight the difficulties of pursuing an intensive regimen in diabetic patients. Considering the evidence supporting the central position of hyperglycaemia in the development of diabetic vascular complications, it seems likely that clinicians will continue to encourage their patients to aim for steady improvements in glycaemic control to reduce the risk of diabetic complications, such as DN.

### RAAS Inhibition: an Old Favourite

The management of hypertension in DN is considered to be an important component in the clinical management of patients. Evidence for this was seen in the UKPDS trial where decreases in systolic blood pressure were associated with a decreased incidence of diabetic complications [66]. Inhibitors of the RAAS, including angiotensin-converting enzyme inhibitors (ACE-I) and angiotensin receptor blockers (ARB), are currently the first-line therapies for DN [69]. The positive effects of ARBs were highlighted by the RENAAL (Reduction of Endpoints in NIDDM with the Angiotensin II Antagonist Losartan) [70] and IRMA-2 (Irbesartan in Patients with Type 2 Diabetes and Microalbuminuria) [71] trials. Moreover, the DETAIL (Diabetics Exposed to Telmisartan and Enalapril) trial confirmed the consensus that both ACE-Is and ARBs have equal efficacy in DN [72]. It is accepted that these agents provide benefits beyond mere blood pressure control, as evidenced by reductions in microalbuminuria [73]. By reducing intra-glomerular pressure, ACE-Is and ARBs have long-term effects on renal function even after cessation, suggesting that these anti-hypertensive drugs normalise some aspects of DN pathology [70]. Whilst these agents were initially used in all DN patients, recent guidelines recommend limiting their prescription to patients with proteinuria > 300 mg/day [74]. Despite representing the current treatment of choice, these drugs merely slow DN progression. Additionally, traditional approaches to inhibit RAAS are limited by an increased risk of hyperkalaemia in patients with advanced disease. As such, there has been a concerted effort to optimise RAAS inhibition, whilst limiting conceivable adverse effects.

Recently, researchers questioned whether or not dual RAAS blockade could provide additional nephroprotective effects. In the ONTARGET (Ongoing Telmisartan Along and in Combination with Ramipril Global Endpoint Trial) study, combining the ARB, telmisartan, with the ACE-I, ramipril, did not improve proteinuria levels and, more importantly, was associated with increased rates of hyperkalaemia [75]. Similarly, the VA NEPHRON-D (Veterans Affairs Nephropathy in Diabetes) trial destroyed any hopes for dual blockade with an ACE-I and an ARB by confirming that this approach offered no significant benefit in the primary endpoints of renal disease progression or death [76]. In fact, the trial documented increased risks of serious adverse effects, including hyperkalaemia and acute kidney injury [76]. Despite considerable evidence that dual blockade should be avoided due to adverse side effects, there is an indication that an increasing proportion of patients is being managed in this way [77]. However, a recent glimmer of hope emerged following the development of novel potassium-binding drugs, including the oral non-absorbed polymer, patiromer. When used in combination with RAAS inhibition, only 15% of stage 3/4 CKD patients developed hyperkalaemia compared to 60% among the placebo group [78]. Direct renin inhibition was considered as another approach to optimise RAAS modulation. Initial results from the AVOID trial were promising, as the addition of a direct renin inhibitor, aliskiren, to losartan therapy resulted in a 20% reduction in the urinary albumin to creatinine ratio [79]. However, in a similar manner to dual RAAS blockade, the larger ALTITUDE study was terminated after 2.7 years due to the absence of change in renal outcomes and excess adverse effect risk in the aliskiren arm of the trial [80]. As it stands, clinicians are currently limited to prescribing only one RAAS-targeting agent for DN patients. Taken together, it seems that conventional RAAS blockade is reaching its limits in terms of DN patient benefit.

## Novel Therapeutic Approaches

### Anti-diabetic Drugs: Unanticipated Benefits

Whilst researchers are often driven to develop new targets and novel therapies, an important part of medicine involves recognising when the solution is already within our grasp. As such, a number of currently available anti-diabetic drugs have shown promise in the treatment of DN. One particularly promising group is the sodium-glucose cotransporter-2 (SGLT2) inhibitors, such as empagliflozin. Beyond blood glucose control, these drugs possess potent renoprotective effects. A recent study by Wang et al. observed increased SGLT2 expression in renal biopsies from diabetic patients and that inhibition prevented DN development in db/db mice, a leptin-deficient type 2 diabetic model [81]. Furthermore, the cardiovascular protection offered by these drugs has successfully translated into clinical studies, as noted by the EMPA-REG OUTCOME trial [82]. Despite being prescribed for type 2 DM, SGLT2 inhibitors may afford further renoprotection to younger type 1 DM patients, as demonstrated when empagliflozin was added to insulin therapy [83]. The ability of these drugs to target advanced kidney disease may, however, be questionable, as glucose-modulating effects lessened with decreasing eGFR. As such, the dedicated renal trial, CREDENCE [NCT02065791], should answer important questions regarding the potential of SGLT2 inhibitors to treat DN. Beyond SGLT2 inhibitors, clinical trials have suggested that glucagon-like peptide-1 (GLP-1) receptor agonists and dipeptidyl peptidase-4 (DPP-4) inhibitors can improve renal function, possibly independent of their anti-glycaemic properties [84, 85]. These findings suggest that clinicians already have access to a wealth of established drugs that could potentially improve renal deterioration in DN patients.

### Beyond the Limits of RAAS Inhibition

Under physiological conditions, two opposing arms of the RAAS remain in relative equilibrium: the pressor arm (ACE-I/angiotensin II) and the depressor arm (ACE-2/Ang-(1–7)). There is increasing evidence that modulating the counter regulatory depressor arm can attenuate kidney damage. Chou et al. showed that hyperglycaemia lowered ACE-2 levels in rat proximal tubular cells [86]. Additionally, the positive effects of human recombinant ACE-2 in diabetic Akita mice were demonstrated by Oudit et al. with attenuated matrix expansion and blood pressure normalisation [87]. Moreover, whilst the Ang-(1–7) heptapeptide has been shown to alter various disease indices, it is evident that its short half-life will need to be improved to facilitate the development of clinically relevant formulations [88, 89]. Despite the apparent ceiling in terms of the RAAS, new developments suggest that progress can still be made in improving the efficacy of targeting this system in DN.

### Oxidative Stress: Dampening the Destruction

Oxidative stress has emerged as an important novel target for DN, as imbalanced redox signalling resulting from the excessive generation of reactive oxygen species (ROS) exacerbates renal injury. Unfortunately, non-selective inhibition of oxidative stress has so far failed as a DN therapy, possibly due to unintentional interference with physiological ROS activity and the increasingly apparent complexity of redox signalling. More effective approaches may involve intensifying the effects of endogenous scavenging molecules, e.g. nuclear factor erythroid 2-like 2 (Nrf2) [90]. The potential to target Nrf2 was highlighted by Zheng et al. who showed that the Nrf2 agonists, sulphoraphane and cinnamic aldehyde, reduced oxidative stress and minimised pathological changes in the glomeruli of STZ-induced mice [91]. Clinical data from the BEAM (Bardoxolone Methyl Treatment: Renal Function in CKD/Type 2 Diabetes) trial supported the efficacy of Nrf2 activation, since bardoxylone methyl was associated with improved renal function in patients with Type 2 DM [92]. The subsequent BEACON trial, however, was terminated prematurely due to an increased risk of cardiovascular events [93]. Another option to target this pathway may include the use of drugs with known and well-characterised safety profiles, such as the tetracycline antibiotic, minocycline, which stabilises Nrf2 and decreases oxidative stress [94].

Selective targeting of NADPH oxidase (NOX) enzymes, which are key modulators of the cellular redox state, may also hold therapeutic potential in DN. For example, Gorin et al. first showed that inhibiting NOX4, the most abundant renal isoform, with anti-sense oligonucleotides reduced glomerular expansion in diabetic rats [95]. Recent research also highlighted the ability of a dual NOX1/NOX4 inhibitor, GKT137831, to modulate renal pathology [96, 97]. Specifically, Gorin et al. reported that GKT137831 suppressed diabetes-induced NOX activity and superoxide generation whilst reducing mesangial expansion in OVE26 mice [96]. More recently, Grey et al. demonstrated that delayed intervention with GKT137831 could effectively downregulate pro-inflammatory pathways in animal models with established DN [97]. Interestingly, additional atheroprotection was observed with low doses but was absent with higher concentrations of the drug. Therefore, it seems that the effects of GKT137831 are both dose- and tissue-dependent. Beyond this, a novel pan-NOX inhibitor, APX-115, was shown to have similar or superior efficacy to GKT137831 in DN [98]. This compound reduced both plasma creatinine levels and urinary albumin excretion in db/db obese/diabetic mice [98]. Taken together, whilst these results appear promising, there remains a need to determine whether or not these NOX inhibitors can improve patient endpoints in large, randomised DN clinical trials.

### Inflammation: Calming the Storm

In light of the intrinsic link between inflammation and hyperglycaemia, the ability to modulate key inflammatory cascades appears attractive for DN therapy. For instance, monocyte chemoattractant protein-1 (MCP-1) has emerged as a potential therapeutic target. MCP-1-mediated macrophage accumulation was subsequently linked to progressive functional deterioration [99]. Pre-clinical research has correspondingly highlighted the potential benefits of MCP-1 targeting, showing that it reduced diabetic glomerulosclerosis [100]. These positive effects were hinted at in a number of clinical studies, which showed that two agents targeting the MCP-1 axis, emapticap [101] and CCX140-B [102], decreased albuminuria levels in type 2 DM patients.

Conflicting evidence has also emerged with regard to anti-inflammatory agents. Despite the central role of TGF-β in propagating inflammatory renal damage, clinical trials of TGF-β inhibitors reported underwhelming results. As such, the promise of anti-TGF-β_1_ therapy failed to materialise when a phase 2 study showed that there were no positive effects on DN progression [103]. The multi-functional capabilities of TGF-β are increasingly being unveiled. In this way, research has challenged the previously well-accepted pathogenic role of TGF-β by illustrating its potent anti-inflammatory [104] and anti-apoptotic potential [105]. Significantly, a recent paper published in JASN demonstrated that a dual specificity anti-TGF-β antibody could reduce renal lesions when specifically targeted to the extracellular matrix of the kidney [106].

The T cell modulator, abatacept, which is currently licenced for rheumatoid arthritis, failed to preserve renal function in STZ-induced mice, as reflected by a failure to alter the albumin to creatinine ratio [107]. On the other hand, the potential to repurpose an old drug for DN has proved promising with regard to the methyxanthine derivative, pentoxifylline. The ability of this approved claudication therapy to inhibit TNF-α may have contributed to the promising results observed during the PREDIAN trial, where pentoxifylline caused significant reductions in urinary albumin excretion in DN patients [108]. Taken together, these studies suggest that specific elements of the immune system drive DN pathology and as such, translational efforts will need to focus toward their identification and targeting. It should also be noted that albuminuria may not be the most appropriate surrogate marker of efficacy for anti-inflammatory agents. As such, until novel biomarkers can be identified, future research will need to consider the evaluation of hard renal endpoints to monitor patient response to therapy.

### Renal Fibrosis: an Emerging Target

Beyond inflammation and oxidative stress, many groups have proposed that targeting renal fibrosis may be an effective way to further slow functional deterioration in DN. The TGF-β inhibitor, pirfenidone, has shown anti-fibrotic and renoprotective capabilities [109], but its use was associated with gastrointestinal side effects [110]. Interestingly, a phase I trial of anti-connective tissue growth factor (CTGF) monoclonal antibodies demonstrated an ability to decrease the urinary albumin to creatinine ratio [111] but following the termination of a phase II trial, it seems that this strategy is no longer being pursued. Of interest, the DPP-4 inhibitor, linagliptin, demonstrated notable anti-fibrotic effects via the inhibition of TGF-β2-induced EndoMT in a type 1 diabetic mouse model [112]. Reductions in the levels of gremlin 1 (GREM1) have also been shown to attenuate diabetic and other forms of renal fibrosis [113–115]. Other options to target renal fibrosis have recently included targeting homeodomain-interacting protein kinase 2 (HIPK2) [116] or SMAD7 gene transfer [117]. MicroRNA modulation has shown significant promise, as the inhibition of mir-192 successfully repressed renal fibrosis in DN [118]. Mir-103b inhibition also improved renal tubulointerstitial fibrosis by suppressing Snail-induced EMT [119]. Vitamin D deficiency has been linked to an increased severity of structural renal alterations [120]. In a study by Tian et al., administration of active vitamin D attenuated renal fibrosis and protected renal function [121], making this approach a potential future consideration in the management of DN patients. Many of these therapies remain in the pre-clinical phase, and their efficacy in human patients has yet to be demonstrated.

### Harnessing Cellular Defences

An alternative approach to DN treatment could revolve around the use of agents to harness the body’s cytoprotective pathways. Thus, instead of blocking disease-driving molecules, it may be more fruitful to focus on those agents that can mobilise our innate molecular defences. Such approaches may be able to circumvent the damaging effects of glucotoxicity. One relevant mechanism is the nutrient-sensitive process of autophagy, which is downregulated in DN [122]. Autophagy is a tightly regulated lysosomal process that results in the degradation of unwanted cytoplasmic constituents [123] and is thus responsible for maintaining intra-cellular homeostasis. Moreover, autophagy is activated in response to cellular stresses, including nutrient deprivation, oxidative stress and metabolic abnormalities. Evidence suggests that in DN, autophagic systems become insufficient and that deficient autophagy has the potential to accelerate renal fibrosis [124]. Importantly, key features of DN were recapitulated in animal models via enhanced activation of the autophagy-inhibiting gene, mechanistic target of rapamycin kinase (mTOR) [125]. Moreover, levels of the mitochondrial protector, sirtuin 1, were significantly reduced in the diabetic kidney [126].

Considering the proposed importance of autophagy in the maintenance of renal homeostasis, the complexities of this mechanism remain incompletely understood. Whilst this process is impaired in both constituent cells of the glomeruli and the renal tubules, its impact on podocyte destruction is particularly interesting. Podocytes are terminally differentiated epithelial cells, which form an important layer in the molecular sieve of the glomerular basement membrane. Their role in DN pathogenesis was confirmed in a landmark publication by Pagtalunan et al., which showed that a reduction in podocyte number was positively associated with albuminuria [127]. Given their inability to repair following damage, these cells demonstrate high basal autophagic activity under physiological conditions. A more recent study reported that podocyte-specific autophagy-deficient diabetic mice developed significant proteinuria, whilst also confirming the lack of autophagy in renal biopsy tissue, which displayed intra-cellular accumulation of p62, a specific autophagy target [128]. Furthermore, another group demonstrated that hyperglycaemia-induced overexpression of the permeability-inducing molecule vascular endothelial growth factor (VEGF) could be enhanced by the autophagy inhibitor, 3-methyladenine [129]. These findings were in line with previous research demonstrating that this autophagy-driven phenomenon was sensitive to changing levels of ROS [129]. However, caution must be exercised when interpreting and undertaking autophagy-focused studies so as to avoid the extrapolation of findings regarding the amount of autophagy-produced substrates to variations in autophagy instability among cells.

As such, the ability to modulate autophagy may provide an exciting mechanism to reverse DN pathology. Indeed, calorie restriction in diabetic rats proved to regulate disordered autophagy by upregulating sirtuin 1 [130] and by suppressing mTOR [131]. Given the widely accepted health-prolonging benefits of calorie restriction, the adherence of patients to intense dietary modifications may be a useful additive approach. However, the degree of calorie restriction remains poorly defined and implementation of such radical health behaviours may ultimately be difficult to implement. Alternatively, using compounds to modulate these nutrient-sensitive pathways may be a more feasible strategy. Such agents could include the anti-diabetic drug metformin [132] or the herbal compound paeoniflorin [133]. In particular, modulating the expression of AMP-activated protein kinase (AMPK), an autophagy activator and target of metformin, has proven worthwhile. Kim et al. found that phosphorylation of AMPK by resveratrol prevented renal lipotoxicity and glucotoxicity [134]. In addition, Lee et al. demonstrated that pharmacological activation of AMPK successfully attenuated renal hypertrophy in diabetic rats [135]. Moreover, the macrolide compound, rapamycin, has proven capable of inhibiting the mTORC1 pathway and its potent ability to modulate DN pathology was demonstrated in various animal models [136, 137]. To date, the potential targeting of this intricate cellular defence mechanism appears promising. However, this therapeutic strategy is still in its infancy and as such, further research is required to inform more sophisticated approaches to reactivate autophagy.

### Stem Cells: the Emerging Frontier

Recent years have seen increasing attempts to move the field of stem cell biology into the clinical environment. However, the strategies taken to harness the therapeutic potential of stem cells have proven to be challenging. Whilst the clinical use of embryonic stem cells is hampered by a string of ethical concerns, the emergence of induced pluripotent stem cells (iPSCs) has provided an attractive alternative. A number of groups have reported the generation of renal subtypes from iPSCs [138–140]. For example, Lam et al. produced cells characteristic of intermediate mesoderm, which subsequently expressed characteristic proximal tubular markers [140]. It should, however, be pointed out that iPSC-derived populations display significant phenotypic variability. Beyond the difficulties of generating pure cell populations, methods for renal lineage differentiation require additional optimisation in order to produce the high-quality cell yield needed for therapeutic purposes.

Considering that the kidney is essentially a mesodermal organ, focus has understandably shifted to mesenchymal stem cells (MSCs), which are multi-potent bone marrow-derived cells and connective tissue progenitors. Interestingly, Wang et al. showed that intra-arterial administration of MSCs to type 1 diabetic Sprague Dawley rats reduced podocyte effacement and prevented the loss of glomerular nephrin and podocin [141]. Whilst this treatment did not alter blood glucose levels, Ezquer et al. reported that MSC-treated diabetic mice demonstrated improved glycaemic levels within 1 week and that remarkably, euglycaemia was achieved within 1 month post-treatment [142].

As well as their regenerative ability, MSCs possess potent immunomodulatory functions and are capable of secreting various signalling molecules by which, among other actions, they are able to antagonise TGF-β signalling [143]. Indeed, these cells are known to exert many of their effects through paracrine signalling. Moreover, exosomes contained in MSC-conditioned media were recently shown to be key orchestrators of their therapeutic actions [144]. Taken together, it is evident that MSCs hold significant therapeutic potential; the ongoing EU-funded NEPHSTROM trial [NCT02585622] will provide essential safety information for allogenic bone marrow-derived MSC treatment in DN patients.

## Conclusions and Perspectives

DN is a serious complication that develops in approximately one third of diabetic patients. Unfortunately, despite the obvious benefits derived from managing hyperglycaemia and haemodynamic dysregulation, many patients progress to ESRD requiring dialysis or transplant. Although there are continued advancements in the field, the underlying molecular mechanisms of DN have yet to be completely elucidated. Even though hyperglycaemia-induced TGF-β upregulation has emerged as a central pathogenic mechanism in DN, research has highlighted the contributions of additional pleiotropic cellular events. These changes are set against the background of a multi-layered cellular communication system within the kidney. It has therefore been proposed that DN cannot be considered as a simple ‘microvascular’ disease. Future efforts to refine our understanding of DN pathophysiology will be necessary to resolve the current debates, determine the relative importance of newly identified pathogenic mechanisms and prioritise novel therapeutic targets.

**Fig. 2:**
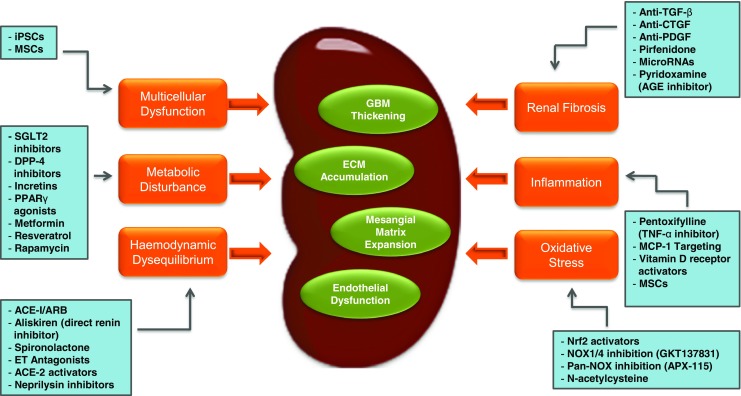
Potential target pathways and therapeutic agents currently under investigation for the treatment of diabetic nephropathy: iPSCs induced pluripotent stem cells, MSCs mesenchymal stem cells, SGLT2 sodium-glucose cotransporter-2, DPP-4 dipeptidyl peptidase-4, PPARγ peroxisome proliferator-activated receptor gamma, ACE-I/ARB angiotensin-converting enzyme inhibitor, angiotensin receptor blocker, ET endothelin receptor, ACE-2 angiotensin-converting enzyme 2, GBM glomerular basement membrane, ECM extracellular matrix, TGF-β tissue growth factor-beta, CTGF connective tissue growth factor, PDGF platelet-derived growth factor, AGE advanced glycation end products, MCP-1 monocyte chemoattractant protein-1, Nrf2 nuclear factor erythroid 2-like 2

Whilst the complex nature of DN is likely responsible for many of the obstacles that are preventing the successful translation of novel targets into effective DN therapeutics, this complexity has provided researchers with a wealth of pathways available for potential targeting (Fig. 2). Such research should aid in the translation of new DN-targeted therapies that will improve patient outcomes. Oxidative stress, inflammation and fibrosis are pivotal interlocking pathogenic processes that contribute to DN. As such, the ability to target each of these deleterious mechanisms presents an exciting therapeutic avenue. Despite appearing as obvious targets, modulation of these pathways has not been straightforward. Novel approaches, including cellular therapies or the modulation of autophagy, may eventually revolutionise DN treatment. Nevertheless, many challenges remain for researchers in the field of DN. These range from the use of unrepresentative animal models to poorly defined clinical trial inclusion criteria, which make it almost impossible to confirm causality. Additionally, the ability to measure traditional renal endpoints necessitates the use of resource-intensive, long-lasting clinical trials. In this regard, multi-level ‘omics’ studies may help to identify appropriate DN biomarkers. Improved design and execution of clinical trials for DN are urgently needed. Drug safety is another major concern, as the suboptimal compensatory mechanisms in DN patients make them particularly vulnerable to adverse drug effects. Moreover, the need to foresee potential drug interactions will be an important step to improving patient outcomes.

Further to cutting-edge developments in genomic technology, research has pinpointed several key pathways related to DN initiation and progression. The true challenge, however, will be to overcome the inherent heterogeneity of DN to identify novel drug targets that are relevant to large patient numbers. In essence, the successful management of DN will hinge on the implementation of a multi-disciplinary, multi-target approach, in which novel therapeutics are coupled with the traditional management strategies of promoting glucose control, blood pressure management and continued engagement in all-round positive health behaviours. Such complexity dictates that one treatment for DN will not cure all, so more personalised approaches will be required. In essence, it is only with this realisation in mind that medical professionals will be best equipped to manage the DN patient population. Continued investment in basic and clinical researches will be essential to successfully translate exciting pre-clinical data into reliable biomarkers and effective therapeutics for DN. Such work holds the potential to bring about measurable changes in clinical outcomes and to establish an improved quality and quantity of life for the DN patient population.
